# Suitability of the MP1000 system for robot-assisted partial nephrectomy: a multicenter randomized controlled noninferiority trial

**DOI:** 10.1097/JS9.0000000000001166

**Published:** 2024-02-13

**Authors:** Yu Gao, Yang Yang, Shaoxi Niu, Wang He, Jin Tao, Shengjie Guo, Hongzhao Li, Xin Ma, Xing Ai, Jian Huang, Fangjian Zhou, Xuepei Zhang, Xu Zhang

**Affiliations:** a Department of Urology, The Third Medical Centre, Chinese PLA General Hospital; b Medical School of Chinese PLA; c Department of Urology, Sun Yat-Sen Memorial Hospital, Sun Yat-Sen University, Yuexiu District; d Department of Urology, Sun Yat-sen University Cancer Center, Guangzhou; e Department of Urology, The First Affiliated Hospital of Zhengzhou University, No. 1 Jianshe East Road, Zhengzhou, People’s Republic of China

**Keywords:** prognosis, robotic surgery, RAPN

## Abstract

**Purpose:**

This study aimed to compare the safety and effectiveness of the MP1000 surgical system with the da Vinci Si robot system in robot-assisted partial nephrectomy (RAPN) through a prospective, single-blinded, randomized controlled trial.

**Materials and methods:**

A total of 62 patients who were scheduled to undergo RAPN were randomly assigned to either the da Vinci Si robot or MP1000 group. A noninferiority test was conducted with a noninferior intermediate value of 10%. The study compared installation and operation times, estimated blood loss, warm ischemia time, postoperative surgical margin, rate of conversion to open surgery, eGFR level, complications, and other safety indicators between the two groups.

**Results:**

All procedures were successfully completed without the need for conversion to open or laparoscopic surgery, and no major complications were observed during the process. The test of noninferiority was achieved. There were no significant differences in median installation time, operation time, complication rate at 3 months, rate of positive surgical margin, and eGFR level at 3 months between the groups. Additionally, no evidence of recurrence was found on imaging in both groups. No difference in National Aeronautics and Space Administration task load index results for ergonomic considerations. A limitation of this study was its small sample size.

**Conclusions:**

The MP1000 system is a suitable platform for RAPN with safety and effectiveness compared with da Vinci Si system.

## Introduction

Highlights

- Surgeons who are experienced with da Vinci Si found that the components and controls are similar, making it easy to switch systems with a smooth learning curve.
- The MP1000 arms are suspended on a beam above the patient and surgical assistants, minimizing interference.
- The control console of the MP1000 provides surgeons with a glasses-free three-dimensional vision, with high resolution and accurate color rendering, making it ideal for precision surgeries.
- The creators of the MP1000 are currently developing a single-port robotic system. The control console has been designed to work for both systems, making it cost-effective to purchase.
- The MP1000 system is specifically designed for the Chinese population, with surgical instrument sizes tailored to the Chinese figure.
- Some of the costs associated with the MP1000 system surgery may be covered by insurance, reducing patient expenses even further.

As the field of urology gains a deeper understanding of the biological behavior and renal physiology of renal tumors, nephron sparing surgery (NSS) has become the standard treatment for T1a renal tumors and an alternative for some T1b-T2 renal tumors^1,2^. In 2004, the first case of robot-assisted partial nephrectomy (RAPN) was reported by Gettman^3^. The da Vinci robotic surgical system offers unique advantages in deep and fine-manipulation compared to conventional laparoscopic techniques^4^. Additionally, other advantages such as a 3D high-definition visual field imaging system, full-degree-of-freedom joints, and ergonomic control system can help surgeons overcome the limitations of conventional laparoscopy and enable smoother surgeries^5,6^. However, the cost of robotic surgery is a significant concern, as the average cost exceeds $6000, thereby limiting its widespread use^7^. To address this issue, various robotic systems such as the Revo-i system^8,9^ and the Senhance robotic platform^10,11^ have been developed and applied to RAPN. The release of new robotic platforms could lead to potential cost reductions. In China, the self-developed robot platform MP1000 has shown promising results. For RAPN, a multicenter RCT study was conducted in China and is reported as follows.

## Methods

### Design

This multicenter randomized controlled noninferiority trial was conducted from April 2021 to September 2021 at four centers in China. The trial was reported in line with Consolidated Standards of Reporting Trials (CONSORT) Guidelines^12^.

Written informed consent was obtained from all eligible patients. Patients under 75 years of age who were suitable for RAPN were included, while those with a history of abdominal surgery were excluded due to the possibility of severe adhesions. A total of 62 patients were enrolled in the trial and were allocated 1:1 in each group. The random allocation sequence for the randomized block design was generated by a statistician using SAS 9.4. The sequence was then placed in opaque sealed envelopes. When a new patient entered the study, the investigator would open an envelope, revealing the treatment allocation. Both the patients and investigators remained unaware of the treatment allocation until the envelope was opened. The treatment allocation was also concealed from the pathologists and individuals who assessed the outcomes throughout the entire study. All operations were performed by four experienced and high-volume surgeons, with each surgeon having performed more than 800 RAPN operations using standard robotic surgical procedures and have hands-on experience with the MP1000 in relevant animal experiments (over 30 cases of partial nephrectomy performed in pigs). All surgeons participating in the study were adequately trained in standardized surgical techniques. Preoperative, intraoperative, and postoperative data were prospectively collected.

The preoperative data collected included the patient’s age, BMI, sex, preoperative eGFR level, and clinical stage. Intraoperative data included installation time, operation time, estimated blood loss (EBL), intraoperative organic or vascular damage, and safety events. Postoperative data included the visual analog scale (VAS), postoperative complications (Clavien–Dindo), positive surgical margin (PSM), recurrence, and eGFR levels (3 months after the operation). The installation time was defined as the time interval from when the patient cart started moving to the last catheter docking with the corresponding robotic arm. The operation time was defined as the time spent to complete the surgery with the console. The warm ischemia time was defined as the time from blocking to unclamping of the renal vessels.

The study utilized the National Aeronautics and Space Administration task load index (NASA-TLX) for subjective evaluation, specifically the Paper/Pencil Version. To simplify the application process, the original NASA-TLX continuous rating scale (0–100) was modified to a 10-point scale and the weighting process was eliminated. The ratings were then added to estimate global workload. The evaluation of all specimens was conducted by two senior pathologists. In this study, PSM was defined as a portion of the tumor on the inked surface of the renal specimen.

### Procedure

**Figure 1:**
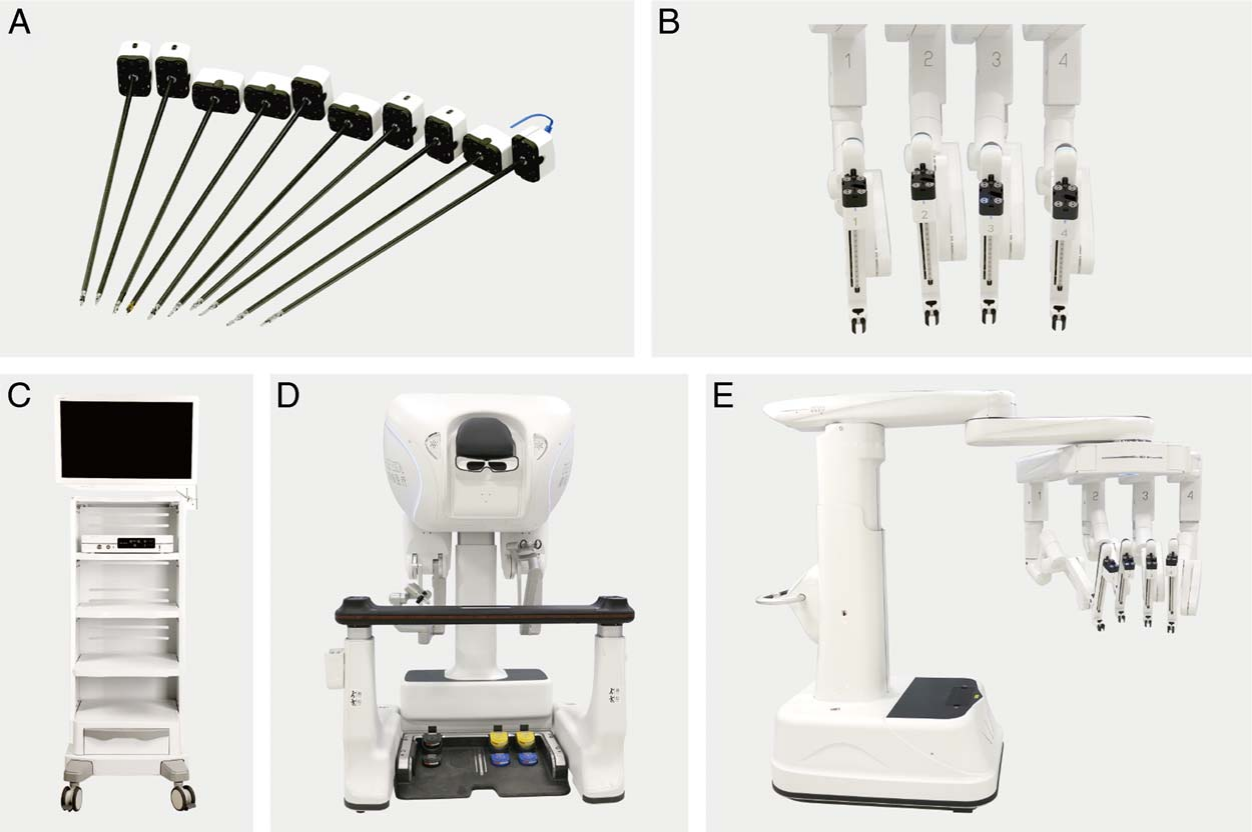
The MP1000 system: (A, B) The cannulas and manipulator arms, (C) The vision cart, (D) The surgeon console, (E) The patient side cart.

**Figure 2:**
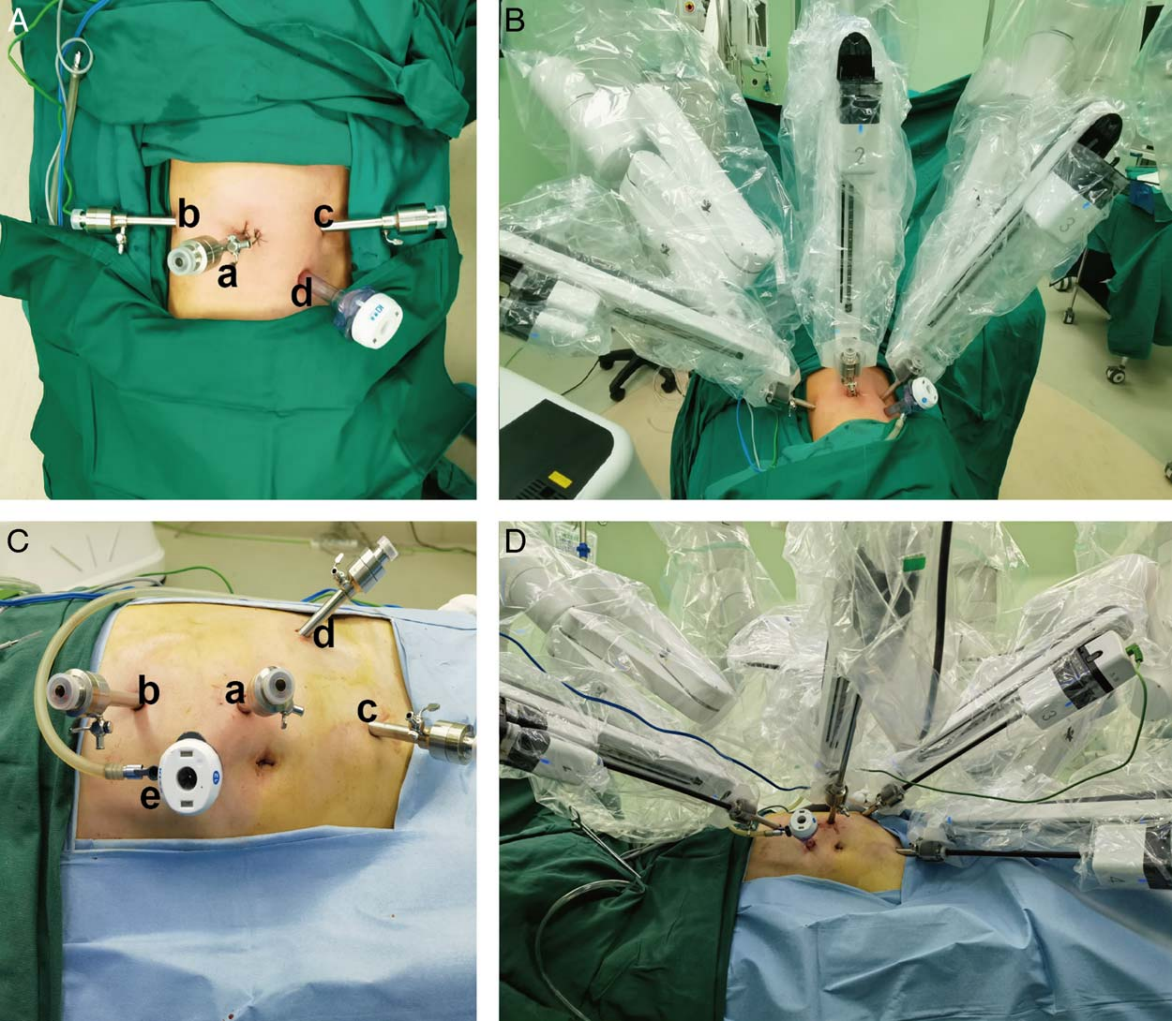
Patient position and port placement: (A, B) Retroperitoneal RAPN with MP1000 system: The patient was secured in the lateral decubitus position with extended flank to expose the space between the costal margin and iliac crest. The first trocar is placed 2 cm above the iliac crest on the midaxillary line for the camera trocar (a). 8 mm second robotic arm trocar inserted at the mid point of the costal margin and iliac crest on the posterior axillary line (b). 8 mm first robotic arm trocar is placed at 1 cm medial to anterior axillary line at the level of second arm trocar (c). The assistant 12 mm trocar is inserted just above the anterior superior iliac spine (d). (C, D) Transperitoneal RAPN with MP1000 system: The patient is secured on the operating table in the 45° extended flank position. The first 12 mm trocar for the robot camera is placed at the 2 cm proximal and lateral to the umbilical (a). The two 8 mm trocar for arm 1 and arm 2 are placed superior and inferolateral to the camera trocar respectively (b, c). The 8 mm trocar for arm 3 is placed just above the pubic tubercle same line to the camera trocar (d). The location of the 12 mm assistant trocar can be altered based on the assistant preference, which can be placed between the arm1 and camera trocar, or at the subumbilical (e).

**Figure 3:**
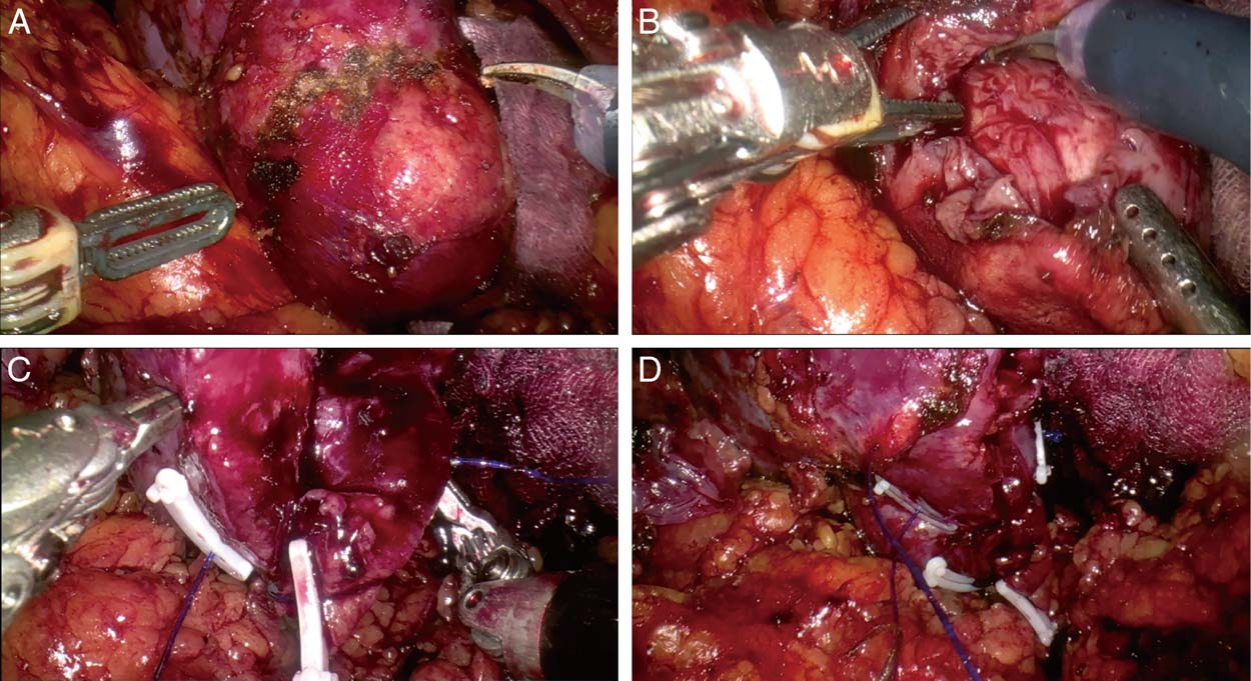
RAPN procedure with the MP1000 system: (A) Exposure and labeling of the renal tumor (B) Complete resection of renal tumor (C, D) Barbed suture closure of the wound.

The MP1000 system consists of a surgeon control console, patient and vision carts, and reusable endoscopic instruments (Fig. 1). The patient cart is equipped with four arms, and a robotic camera can be installed in any arm as needed. The patient’s position and port placement were identical for both systems (Fig. 2). The surgical procedure was consistent across both groups and involved either a transperitoneal or retroperitoneal approach based on tumor location. The warm ischemia method was used, with all renal arteries being clamped using a bulldog clamp. After tumor resection, renorrhaphy was carried out using a two-layer suturing technique, and an early unclamping technique was utilized (Fig. 3).

### Outcomes

The primary outcome of the study was to evaluate the success rate of the operation. Successful operation was defined as 1. Completing the surgery without converting to laparoscopic or open surgery, 2. The WIT was <30 min, 3. The pathological margin was negative. The study also assessed several secondary outcomes, including installation time, operation time, EBL, intraoperative organic or vascular damage, safety events, ergonomics, VAS, postoperative complications, PSM, recurrence rate, and eGFR levels (3 months after the operation). Complications were evaluated using the Clavien–Dindo classification, a commonly used tool in urological procedures^13^. Safety events were defined as linkage interruption between the control console and patient cart, clamping issues, intraoperative robot error alarm, and instrument breakdown or damage.

### Sample size and statistical analysis

The study utilized a randomized controlled trial design to evaluate the effectiveness of the research object. The main evaluation index of the observation was the effective rate. Based on the pre-experimental results, both the MP1000 group and Si group had an effective rate of 0.98. The bilateral α value was set at 0.05 (0.025 on each side) and the power (1-β) was set at 0.8. The sample size ratio between the MP1000 group and Si group was 1:1, with a noninferiority boundary value of 0.1. Accounting for a 10% loss of follow-up and rejection of follow-up, a sample size of 31 per group was determined. Quantitative variables are described as mean, SD, maximum, minimum, median, lower quartile (Q1), and upper quartile (Q3). The *t*-test or Wilcoxon’s test was performed to compare the two groups after testing for normal distribution. Categorical variables are described by cases and percentages; the *χ* ^2^ or Fisher’s exact test was used to compare these. The Wilson method calculated the 95% CI of operative success rates. All data were analyzed using SAS9.4 (SAS Institute), and statistical significance was set at *P*<0.05, unless stated otherwise.

## Results

### Participants

In the clinical trial, five patients from the MP1000 group and three patients from the Si group withdrew due to concerns about tumor control before surgery and opted for radical nephrectomy. All eight patients had clinical stages of T1b. So this study included 26 patients in the MP1000 group and 28 patients in the Si group. Table 1 shows that the demographic characteristics of the study patients were similar between the two groups.

**Table 1:** Demographic characteristics and clinical characteristics.

	MP1000 group	Si group	*P*
Patients, *n* (Missing)	26 (0)	28 (0)	
Sex			
Men, *n* (%)	19 (73.08)	17 (60.71)	0.336
Women, *n* (%)	7 (26.92)	11 (39.29)	
Age (year), mean (SD)	52.12±10.99	56.18±10.36	0.199
Height (cm), mean (SD)	166.02±8.59	166.29±8.66	0.910
Weight (kg), mean (SD)	68.76±10.44	68.58±11.70	0.953
BMI, kg/m^2^, mean (SD)	24.89±2.87	24.68±2.84	0.787
RENAL nephrometry score			
Radius ≤4 cm, *n* (%)	22 (84.62)	24 (85.71)	0.787
4 <Radius ≤7 cm, *n* (%)	4 (15.38)	4 (14.29)	
Exophytic property			
Exophytic >50%, *n* (%)	13 (50.00)	12 (42.86)	0.871
Exophytic <50%, *n* (%)	9 (34.62)	11 (39.29)	
Completely endophytic, *n* (%)	4 (15.38)	5 (17.86)	
Nearness of the deepest portion of the tumor to the collecting system (mm), *n* (%)			
>7	17 (65.38)	12 (42.86)	0.248
4–7	3 (11.54)	6 (21.43)	
<4	6 (23.08)	10 (35.71)	
Anterior/Posterior descriptor, n(%)			
A	9 (34.62)	7 (25.00)	0.462
P	10 (38.46)	9 (32.14)	
X	7 (26.92)	12 (42.86)	
Location relative to the polar line, n(%)			
Upper	12 (46.15)	9 (32.14)	0.493
Middle	8 (30.77)	9 (32.14)	
Lower	6 (23.08)	10 (35.71)	

### Operative variables

For the analysis, all participants were included in their original assigned groups. Table 2 displays the operative variables. All 54 surgeries were completed successfully without any conversions.

**Table 2:** Operative variables.

	MP1000 group	Si group	*P*
Operative success, *n* (%, 95% CI)	26 (100, 84.54–100)	28 (100, 84.54–100)	
Difference in operative success rate (95% CI)	0 (−15.46–15.46)		
Installation time (min), median (Q1, Q3)	19.50 (19.00–21.75)	22.00 (20.00–23.25)	0.068
Operation time (min), median (Q1, Q3)	124.50 (100.00–155.00)	100.00 (81.00–135.00)	0.059
WIT (min), median (Q1, Q3)	18.00 (12.25–24.00)	16.00 (11.61–24.00)	0.068
Estimated blood loss (ml), median (Q1, Q3)	50.00 (20.00–100.00)	25.00 (20.00–50.00)	0.107
Intraoperative organic or vascular damage, *n* (%)	0	0	
Safety events, *n* (%)			
Interruption of connection between console and robot arms and reconnection fail	0	0	
Instruments could not be loosened when clamping tissues	0	0	
Error alarm of robot	0	0	
Instruments failure or damage	0	0	

The MP1000 and control groups did not differ in median installation time (19.50 vs. 22.00 min; *P*=0.068) or median blood loss (50.00 vs. 25.00 ml; *P*=0.107). Additionally, the median operation time (124.50 vs. 100.00 min; *P*=0.059) and WIT (18.00 vs. 16.00 min; *P*=0.068) were found to be comparable between the two groups. Importantly, no intraoperative organic or vascular damage occurred in surgeries for either group, and no safety events were recorded.

### Satisfaction of surgeons

Table 3 shows that the NASA-TLX median scores of mental, physical, and temporal demands as well as of performance, effort, and frustration were, respectively, 6, 6.5, 7, 10.5, 7.5, and 5 for the MP1000 group and 8.5, 8, 7, 11, 7, and 4.5 for the Si group. Differences were not significant.

**Table 3:** Evaluation of ergonomics by NASA-TLX.

Item, median (Q1, Q3)	MP1000 group	Si group	*P*
Mental demand	6.00 (5.00–13.00)	8.50 (5.00–10.25)	0.491
Physical demand	6.50 (5.00–13.50)	8.00 (5.00–9.25)	0.289
Temporal demand	7.00 (6.00–11.00)	7.00 (4.75–9.25)	0.151
Performance	10.50 (7.00–14.00)	11.00 (7.75–15.25)	0.404
Effort	7.50 (6.00–13.00)	7.00 (5.00–9.25)	0.137
Frustration	5.00 (4.00–9.00)	4.50 (3.00–7.00)	0.283

### Intraoperative and perioperative variables

Table 4 displays the complications and follow-up data, revealing that no complications arose in the immediate postoperative period. However, one patient in the MP1000 group required antibiotic treatment for postoperative infection (Clavien–Dindo grade I), while four cases of fever necessitated physical cooling (Clavien–Dindo grade I) in the Si group. The postoperative pathologic tumor stage was similar between the groups (*P*=0.481), and no PSM was detected in either group following the operation. Median VAS score was similar between the two groups (4.00 vs. 3.50 min; *P*=0.724). At the 3-month follow-up, imaging did not show any evidence of recurrence in either group. The median eGFR level (97 vs. 95 ml/min/1.73 m^2^; *P*=0.590) and variation (1.86 vs. 2.18 min; *P*=0.302) were comparable between the two groups. The analysis population for eGFR level variations was patients who received RAPN, and the eGFR level variation was calculated as the eGFR level at 1 day before the operation minus the eGFR level at 3 months after the operation.

**Table 4:** Intraoperative and perioperative variables.

	MP1000 group	Si group	*P*
Postoperative complications, *n* (%)			0.186
Overall	1 (3.85)	4 (14.29)	
Clavien–Dindo grade 1	1 (3.85)	4 (14.29)	
Clavien–Dindo grade 2	0	0	
Clavien–Dindo grade 3	0	0	
PSM, *n* (%)	0	0	
VAS, median (Q1, Q3)	4.00 (3.00–5.00)	3.50 (2.00–5.00)	0.724
Tumor stage, *n* (%)			0.481
pT1, *n* (%)	25 (96.15)	28 (100.00)	
pT2, *n* (%)	1 (3.85)	0 (0.00)	
Recurrence at 3-month follow-up visit, *n* (%)	0	0	
eGFR level (ml/min/1.73 m^2^)			
Preoperation, median (Q1, Q3)	98 (59–117)	97 (54–115)	0.617
3-month postoperation, median (Q1, Q3)	97 (61–119)	95 (51–120)	0.590
Variation, median (%) (Q1, Q3)	1.86 (−2.16–6.59)	2.18 (−5.17–4.33)	0.302

P Indicates pathology.

## Discussion

Over the last century, the field of NSS has seen significant technological advancements, evolving from open surgery to laparoscopic surgery, and finally to robotic surgery^14,15^. Numerous scholars have compared the benefits of RAPN with those of laparoscopic and open surgery, providing evidence of the safety and efficacy of the robotic system^16–18^. In recent years, the da Vinci robotic system was officially approved for urological surgery, but due to its high cost^19^, only a limited number of units in China are currently using the system. In recent years, some institutions have employed various robotic brands for urological surgery, and clinical studies have been conducted^20–24^. Previous research has concentrated on surgeries involving the lower urinary tract, with limited studies on NSS, which is crucial in assessing upper urinary tract surgery. Our department has performed hundreds of urological surgeries covering both the upper and lower urinary tract using the MP1000 system since its introduction in 2020. In order to evaluate the feasibility, safety, and efficiency of the MP1000 system for RAPN, we conducted a multicenter, single-blind, prospective randomized controlled trial, which to our knowledge is the first human clinical trial of its kind.

All operations were completed successfully and the installation time, operation time, and EBL were found to be comparable between the two groups. The complications of both systems were also comparable 3 months after the operation. No safety events or damage to important vascular structures, nerves, or organs occurred due to machine errors or connection interruptions, verifying the safety of MP1000. The clinical data, including sex, age, BMI, tumor stage, VAS, and eGFR levels, were consistent between the groups, indicating that demographic characteristics were matched. Furthermore, no PSM occurred in either group after the operation. All patients were found to have no recurrence within a span of 3 months through imaging examination. This suggests that the early oncology results are comparable. The WIT, which may have an impact on postoperative renal function, was also found to be similar in both groups, further highlighting the safety of using MP1000. It is worth noting that the surgical technique required for this procedure involves delicate manipulations such as clamping, resecting, ligating, suturing, and anastomosing, which require technical expertise. Therefore, the MP1000 system may be suitable for a wider range of urological procedures, and a further study is warranted to explore this possibility.

The MP1000 system has several valuable features. Surgeons who are experienced with da Vinci Si found that the components and controls are similar, making it easy to switch systems with a smooth learning curve. Additionally, the MP1000 arms are suspended on a beam above the patient and surgical assistants, minimizing interference. The control console of the MP1000 provides surgeons with a glasses-free three-dimensional vision, with high resolution and accurate color rendering, making it ideal for precision surgeries. Given the smaller physique of Chinese people compared to Westerners, there may be instances where the robotic arms interfere with each other. However, the MP1000 distinguishes itself from other domestic robots by incorporating a fourth mechanical arm that applies tension while grasping tissue, thereby enhancing the overall smoothness of the operation process. This makes communication with the robot supplier more convenient for the surgeon, and allows for more efficient feedback on any problems that may arise during the operation, ultimately leading to a smoother surgical process in the future. These unique features have been evaluated for their ergonomic benefits, even with surgeons who lack experience with the platform. In our clinical trial, four surgeons demonstrated a high level of skill in manipulating the surgical system after undergoing standardized animal model training. Moreover, the creators of the MP1000 are currently developing a single-port robotic system. The control console has been designed to work for both systems, making it cost-effective to purchase. In mainland China, hospitals typically spend $3 million to install a da Vinci Si robot system and charge $6000 per patient for robot-assisted surgery. As more surgical robotic systems are developed and introduced to the market, competition will increase, leading to better technology and lower prices. In our clinical trial, patients were given free access to the MP1000 system. Furthermore, insurance may cover some of the costs related to MP1000 system surgery after the trial, thereby reducing patient expenses even more. So, the cost will be reduced by over 50% compared to the da Vinci Si robot system.

Several disadvantages of MP1000 were identified. Firstly, surgeons required additional training to acclimate themselves to the movement speed of the arms and the clamping force of the graspers. Although the mean operation time for MP1000 was longer than that for da Vinci Si, this difference was not statistically significant. Secondly, MP1000 includes a safety feature that can detect when the surgeon leaves the control console and stops the robotic arms. However, this feature was inadvertently activated in some cases, even when surgeons were still maneuvering the robot. This highlights the need for software updates to improve the safety feature of MP1000.

Our study has some limitations, primarily the small sample size of only 62 patients. Despite this, our data indicates a 100% success rate in the MP1000 group with no serious complications observed. Future studies with larger sample sizes can be conducted to build upon these findings. Additionally, two crucial metrics for evaluating surgical robot systems: grip strength and reaction speed were not analyzed and our 3-month follow-up period may not be sufficient to fully assess long-term results such as tumor recurrence and eGFR levels. To address this, we plan to conduct future studies with longer follow-up periods and with more metrics to better understand the tumor control and postoperative renal function of patients after RAPN with the MP1000 system. Furthermore, we will also evaluate the cost-effectiveness of both systems once the free period of the MP1000 system has ended.

In conclusion, MP1000 is a viable option for RAPN, offering comparable levels of safety and effectiveness as the da Vinci Si system.

## Ethical approval

Our study was approved by the local ethics committee and performed in accordance with the ethical standards of the institutional research committee. This article does not contain any studies with animals performed by any of the authors.

## Informed consent

Informed consent was obtained from all individual participants included in the study.

## Sources of funding

This work was financially supported by the National key research and development program (2022YFB4701700).

## Author contribution

Y.G., Y.Y., S.N., W.H., J.T., S.G., H.L., X.M., and J.H.: contributed to performing the RCT, statistical analysis, article writing and editing; F.Z., X.Z., and X.Z.: contributed to project design, article writing, and editing.

## Conflict of interest disclosure

The authors declare that they have no conflicts of interest.

## Research registration unique identifying number (UIN)

ChiCTR2100045537.

## Guarantor

Xu Zhang.

## Data availability statement

All relevant data are within the manuscript and its additional files.

## Provenance and peer review

Not applicable.
